# Radiation-induced neuroinflammation: a potential protective role for poly(ADP-ribose) polymerase inhibitors?

**DOI:** 10.1093/noajnl/vdab190

**Published:** 2022-01-06

**Authors:** Rodrigo Gutierrez-Quintana, David J Walker, Kaye J Williams, Duncan M Forster, Anthony J Chalmers

**Affiliations:** 1 Institute of Cancer Sciences, College of Medical Veterinary and Life Sciences, University of Glasgow, Glasgow, UK; 2 Division of Pharmacy and Optometry, School of Health Sciences, Manchester Cancer Research Centre, Faculty of Biology, Medicine and Health, The University of Manchester, Manchester, UK; 3 Division of Informatics, Imaging and Data Sciences, Manchester Molecular Imaging Centre, The University of Manchester, Manchester, UK

**Keywords:** DNA damage, glioblastoma, microglia, neuroinflammation, neuroprotection, radiation therapy

## Abstract

Radiotherapy (RT) plays a fundamental role in the treatment of glioblastoma (GBM). GBM are notoriously invasive and harbor a subpopulation of cells with stem-like features which exhibit upregulation of the DNA damage response (DDR) and are radioresistant. High radiation doses are therefore delivered to large brain volumes and are known to extend survival but also cause delayed toxicity with 50%–90% of patients developing neurocognitive dysfunction. Emerging evidence identifies neuroinflammation as a critical mediator of the adverse effects of RT on cognitive function. In addition to its well-established role in promoting repair of radiation-induced DNA damage, activation of poly(ADP-ribose) polymerase (PARP) can exacerbate neuroinflammation by promoting secretion of inflammatory mediators. Therefore, PARP represents an intriguing mechanistic link between radiation-induced activation of the DDR and subsequent neuroinflammation. PARP inhibitors (PARPi) have emerged as promising new agents for GBM when given in combination with RT, with multiple preclinical studies demonstrating radiosensitizing effects and at least 3 compounds being evaluated in clinical trials. We propose that concomitant use of PARPi could reduce radiation-induced neuroinflammation and reduce the severity of radiation-induced cognitive dysfunction while at the same time improving tumor control by enhancing radiosensitivity.

Radiotherapy (RT) plays a fundamental role in the treatment of patients with brain metastases and primary central nervous system (CNS) tumors, extending survival for many of these patients. Of the primary malignant brain tumors, glioblastoma (GBM) is the most common in adults and carries a very poor prognosis (median survival of 12–18 months). GBM are notoriously invasive, infiltrating the brain parenchyma extensively, so high doses of RT (typically 60 Gy) are often delivered to large volumes of brain in order to delay recurrence and extend survival.^1^ The irradiated volume inevitably includes normal, functioning brain tissue, and brain RT frequently causes devastating effects on brain function. They can be divided into acute, subacute, and late effects, of which the late effects have the greatest impact. These arise months or even years after treatment and in many cases culminate in irreversible cognitive decline. While adverse neurocognitive effects of RT are common, the specific impact of RT on quality of life in long-term survivors of GBM has not been well characterized. Predicting the clinical impact of RT is complicated since both acute and late effects of RT on quality of life are context dependent, being influenced by numerous radiobiological (RT dose, volume, timing, and duration), physiological (preexisting brain function), and patient-related factors (age, sex, and comorbidities). Although RT remains the most effective nonsurgical treatment modality for GBM, its efficacy is confounded by the innate and adaptive radioresistance of these tumors which is reflected in their inevitable recurrence.

The fundamental rationale for using RT in cancer treatment is based on the rapidly proliferating phenotype of cancer cells which, in addition to defects in the DNA damage response (DDR), makes them sensitive to fractionated courses of radiation. RT damages cellular DNA by causing single strand breaks (SSBs) and double strand breaks (DSBs). This initiates the DDR, an intricate signaling network of proteins that interrupts cell cycle progression and facilitates DNA repair. Failure to repair DNA breaks can result in cell death either through apoptosis or via mitotic catastrophe, the former being more prevalent in nonmalignant cells and the latter predominating in cancer cells.^2^ A critical subpopulation of GBM cells that retains stem cell characteristics exhibits marked upregulation of the DDR, which renders them resistant to DNA damaging treatments including RT.^3^

Different cell populations within the normal brain also exhibit markedly different levels of radiation sensitivity. Neural stem cells and undifferentiated neural progenitor cells (NPCs) are highly radiosensitive, undergoing apoptosis, proliferative arrest, and premature differentiation into other neural cell types in response to relatively low radiation doses.^4^ As such, it is hypothesized that the neurogenic niches in the brain are the most susceptible to radiation-induced DNA damage and play key roles in the pathogenesis of neurotoxicity and associated cognitive deficits.

Beyond this well-documented phenomenon, the precise mechanisms of RT-induced neurotoxicity are still being investigated and appear to be multiple and complex. Recently, emerging evidence cites radiation-induced neuroinflammation as a critical predictor of the long-term effects of RT on cognitive decline.^5^ Neuroinflammation is the inflammatory response within the CNS and is critical in the resolution of cellular damage and tissue injury upon exposure to a variety of noxious stimuli. The magnitude and duration of the neuroinflammatory response, and hence its functional impact, are strongly dependent upon the nature of the stimulus with potential for both positive (axonal regeneration, tissue repair) and negative effects (neuronal damage, cognitive impairment, anxiety/depression) on brain physiology and behavior. In the context of brain RT, the general consensus is that radiation induces neuroinflammation through activation of microglia, astrocytes, and endothelial cells in response to neural cell damage.^5^ The microglia-mediated neuroinflammation that ensues is similar in nature to that observed in a variety of neurological diseases including Alzheimer’s disease (AD), Parkinson’s disease (PD), cerebral ischemia, multiple sclerosis (MS), and amyotrophic lateral sclerosis.^6^

One of the hallmarks of both the DDR and neuroinflammation is activation of the poly(ADP-ribose) polymerase (PARP) family of proteins. PARP proteins are well characterized for their integral role in the DDR, primarily initiating base excision repair (BER) in response to SSBs. Binding of PARP-1 to DNA breaks activates its catalytic function, which is to generate long, branching chains of poly(ADP-ribose) (PAR) on itself and a variety of target proteins. These modifications stimulate recruitment of BER-associated proteins and dissociation of PARP-1 from the damaged sites, promoting DNA repair and cell survival.^7^ To a lesser extent, PARP proteins also interact with kinases such as ataxia–telangiectasia mutated (ATM), nibrin (NBS1), and DNA-dependent protein kinase catalytic subunit (DNA-PKcs) to mediate DSB repair through the homologous recombination (HR) and nonhomologous end joining (NHEJ) repair pathways.^7^

The mechanisms linking PARP activity to neuroinflammation are complex and less well characterized than its DDR functions. Accumulating evidence implicates PARP-mediated secretion of inflammatory mediators from damaged immune cells as the primary mechanism in multiple CNS diseases, including stoke, brain trauma, and neurodegenerative processes.^8–10^ However, this role for PARP has not been studied in the context of radiation-induced neuroinflammation. PARP hyperactivation is implicated in several diseases sharing inflammatory/immune-mediated pathways including those associated with cancer progression and neurodegeneration.^11^ Therefore, PARP activity represents an intriguing mechanistic link between radiation-induced activation of the DDR and subsequent neuroinflammation.

Despite the relative lack of mechanistic understanding, there is extensive data showing that pharmacological inhibition or genetic depletion of PARP exerts beneficial effects by alleviating neuroinflammation in several animal models of inflammatory CNS conditions including stroke, traumatic brain injury, meningitis, and experimental autoimmune encephalomyelitis (EAE).^12–15^ Likewise, multiple studies in various preclinical tumor models have demonstrated that PARP inhibitors (PARPi) have modest but consistent radiosensitizing effects; indeed at least 3 PARPi are currently undergoing early phase clinical evaluation in a variety of cancer types including GBM.^16^ While PARPi have been found to exacerbate acute radiation toxicity in rapidly proliferating normal tissues such as the esophageal or oropharyngeal mucosa,^17^ early data indicate that they can be combined with brain RT without adverse effects.^18,19^ Utilizing PARPi in combination with brain RT offers the exciting prospect of sparing normal tissue from radiation-induced neuroinflammation whilst enhancing tumor responses to radiation. However, our current understanding of the suppressive effects of PARP inhibition on neuroinflammation is constrained by the overreliance on the use of animal models harboring PARP genetic defects (KO) and the relative lack of pharmacological PARPi capable of penetrating the brain parenchyma. PARPi utilized in clinical and preclinical studies block the enzymatic function of PARP by competing with its nicotinamide adenine dinucleotide (NAD^+^) substrate at the catalytic domain. This interferes with the DDR by suppressing BER function but also by preventing automodification of PARP thus delaying its release. This phenomenon of “PARP trapping” is increasingly recognized as playing a key mechanistic role in the therapeutic effects of PARPi, and accounts for many of the differences observed between pharmacological inhibition and genetic depletion of PARP.^20^

The aim of this review is to provide an overview of the physiological and clinical impact of radiation-induced neuroinflammation and the specific role of PARP enzymes in this response. Of the many PARP family proteins, PARP-1 is by far the most abundant, accounting for up to 90% of poly(ADP-ribosylation) in most cells studied to date. PARP-2 and PARP-3 are much less abundant but have been shown to make measurable contributions to the DDR. Most studies in the neuroinflammation field have focused on PARP-1. Of note, the vast majority of PARPi currently available are active against both PARP-1 and PARP-2. This review will focus primarily on the role of PARP-1 in the pathophysiology of radiation-induced neuroinflammation. We intend to emphasize the importance of PARP hyperactivation in neuroinflammation and discuss the clinical significance of PARPi both as radiosensitizing agents in GBM cells and as potential mitigators of radiation-induced and inflammatory-mediated neurotoxicity.

## Neuroinflammatory Response to Ionizing Radiation

The cellular and molecular mechanisms involved in radiation-induced brain injury and secondary cognitive dysfunction are beginning to be elucidated. Although vascular abnormalities, demyelination, NPC death, decreased neurogenesis, and direct glial activation play important roles, recent studies have revealed that irradiation (IR) can trigger immune responses within the CNS, leading to chronic neuroinflammation.^5^ Neuroinflammation is a complex process, the nature and extent of which depend on the context, duration, and anatomical location of the primary insult. Although the neuroinflammatory response has evolved to mitigate triggering factors in order to restore and maintain homeostasis, it can also be damaging and may result in adverse physiological, biochemical, and behavioral consequences. This inappropriate response is not well understood but seems to be a consequence of the complex mechanisms controlling microglial activation in different brain regions.

The pathophysiology of the neuroinflammatory response to ionizing radiation involves primary structural damage and secondary effects on cell dysfunction that lead to progressive changes and associated cognitive decline long after the initial injury.^5^ For many years, neuroinflammation was considered a self-contained process, predominantly regulated by astrocytes and resident immune cells such as microglia; this contributed to the dogma that the CNS was an “immune-privileged” organ. However, research in the last few years has clearly demonstrated that this is not the case. Neuroinflammation involves interplay between several resident CNS cell types and peripheral immune cells that migrate into the brain parenchyma through the blood–brain barrier (BBB), the permeability of which is often compromised upon endothelial cell insult and subsequent activation.^5^ These discoveries have stimulated a comprehensive reevaluation of neuroinflammatory responses in neurodegenerative diseases, traumatic brain injury and, to a lesser extent, the pathophysiology of radiation-induced brain injury.

**Figure 1.**
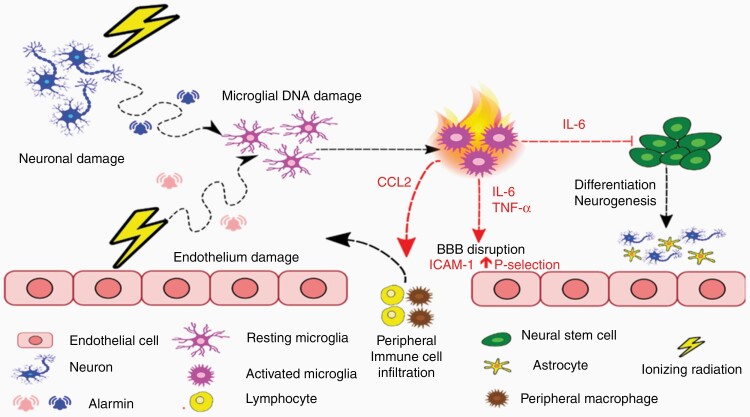
Overview of the impact of ionizing radiation on key CNS cell populations. In response to cellular damage caused by IR, neurons and endothelial cells secrete alarmin molecules (eg, HMGB1) which trigger microglial activation, while radiation-induced DNA damage within microglia themselves also promotes their activation. Proinflammatory molecules secreted by activated microglia, such as IL-6 and TNFα, can prevent neural stem cell differentiation, impacting on neurogenesis and astrocyte development. Inflammatory mediators also stimulate increased expression of endothelial adhesion markers such as ICAM-1 and P-selectin, leading to permeabilization of the blood–brain barrier (BBB) and infiltration of peripheral immune cells into the brain parenchyma. This is further enhanced by the secretion of chemoattractant molecules (CCL2) by activated microglia. CNS, central nervous system; HMGB1, high mobility group box 1; ICAM-1, intercellular adhesion molecule 1; IL-6, interleukin-6; IR, irradiation; TNFα, tumor necrosis factor α.

It is well recognized that IR induces activation of microglia and endothelial cells which together initiate an inflammatory response within the CNS.^21^ Microglia can become activated by alarmins secreted by neurons and endothelial cells that have suffered radiation damage. One well-described example is high mobility group box 1 (HMGB1), expression of which is upregulated following IR exposure and thought to contribute to microglial activation by binding to the toll-like receptor 4.^22^ Another possible mechanism for microglial activation is by direct IR-induced DNA damage causing modification of transcription factors such as nuclear factor κB (NF-κB) and activating protein 1 (AP-1), which control expression of several genes involved in initiating the inflammatory cascade.^23^ This is followed by a surge in inflammatory mediators consisting of cytokines, chemokines, and reactive oxygen species (ROS), which are produced by microglia and astrocytes.^5^ These secretions promote microglial phagocytosis of damaged neural cells and particulate matter, resulting in increased neuronal and progenitor cell death. Inflammatory mediators secreted by microglia and astrocytes, such as interleukin-6 (IL-6), stimulate the endothelium to increase expression of adhesion molecules on the luminal surface, such as intercellular adhesion molecule 1 (ICAM-1) and vascular cell adhesion molecule (VCAM-1), compromising BBB integrity.^24^ Brain microvascular endothelial cells can also be directly activated by IR, increasing expression of adhesion molecules. Leukocytes then attach to endothelial cells and, together with microglia, secrete a panel of matrix metalloproteinases (MMPs) that destabilize the parenchymal basement membrane,^24^ enabling leukocyte migration across the BBB and perivascular spaces into the brain parenchyma to further aggravate neuroinflammation.^25^ Alarmins, such as HMGB1, also contribute to radiation-induced endothelial barrier injury by activation of the mitogen-activated protein kinase signaling pathway.^22^ Among chemokines, CCL2 allows infiltration of peripheral macrophages, altering the brain microenvironment and playing a prominent role in the development of radiation-induced cognitive alterations (Figure 1).^26^

Microglial activation can persist for months after IR, creating a chronic and self-sustaining neuroinflammatory process characterized by a vicious cycle of microglial activation, secretion of proinflammatory agents, neuronal damage, and cell death.^27^ Recently, it has been found that age at the time of IR has an important impact on chronic microglial activation. Using single cell RNA-sequencing, researchers demonstrated that adult mice developed persistent microglial activation after brain IR, whereas juvenile mice (3 weeks old) exhibited an initial dynamic activation microglia that was followed by significant recovery after 1 week.^28,29^ This observation is in keeping with extensive clinical data showing that elderly patients are at increased risk of chronic radiation-induced neurotoxicity.^30^

More specifically, neuroinflammation in the hippocampus has been shown to influence the functional integration of hippocampal neurons. Radiation-induced neuroinflammation disrupts microvascular niches in mouse models, depleting neurogenesis and causing long-term cognitive deficits such as memory loss.^31^ These and other studies have identified that selective inhibition of microglia-mediated neuroinflammation was able to ameliorate radiation-induced cognitive impairment, making it an interesting therapeutic target.^27^

## PARP Activity in Neuroinflammation

PARP-1 is best known for its participation in DNA repair processes, where it is involved chiefly in BER. It also plays a role in alternative NHEJ, which is thought to act as a backup pathway in cells deficient in canonical NHEJ, and interacts with the HR pathway both directly and indirectly via its role in maintenance of DNA replication fork stability.^7^ PARP also contributes to a wide range of cellular and biochemical pathways (reviewed in Weaver and Yang^32^) with increasing evidence indicating a critical role in modulating the inflammatory response both within and without the CNS.

**Figure 2.**
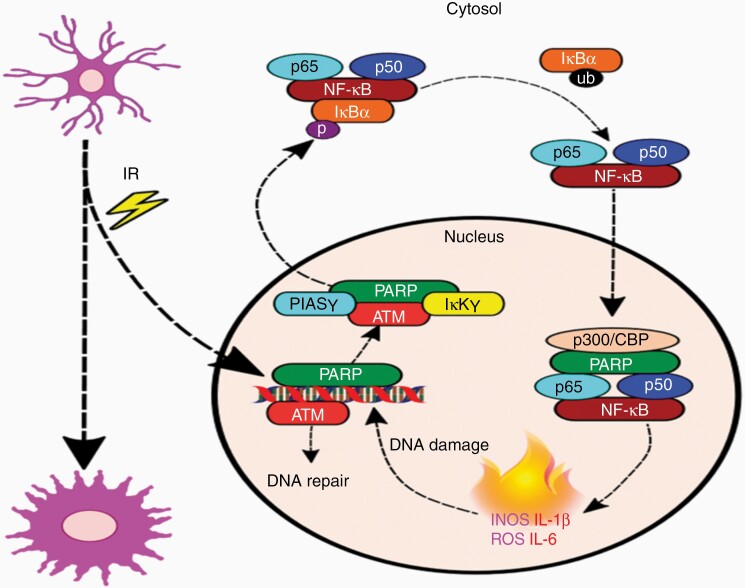
PARP-mediated neuroinflammation in microglia. Following radiation-induced DNA damage, PARP binds to SSBs and recruits BER proteins to induce PARylation and initiate DNA repair. During this process, PARP undergoes automodification which promotes its disassociation from DNA and enables it to form a stable nucleoplasmic protein complex comprised of SUMO1, P1ASγ, NEMO (IKKγ), and ATM. ATM-mediated phosphorylation of NEMO triggers sumoylation of the inactive NF-κB complex in the cytoplasm and subsequent ubiquitination of NEMO from the activated complex. In the nucleus, PARP physically binds to the p65/p50 subunits through p300-CBP histone acetyltransferase, allowing NF-κB-driven transcription of proinflammatory molecules. Unsustainable levels of inflammatory gene expression can lead to reciprocal increases in DNA damage, causing a positive feedback loop that further activates PARP and NF-κB, exacerbating oxidative stress and neuroinflammation. ATM, ataxia–telangiectasia mutated; BER, base excision repair; NF-κB, nuclear factor κB; PARP, poly(ADP-ribose) polymerase; SSBs, single strand breaks.

The most established pathway through which PARP-1 promotes neuroinflammation is via regulation of proinflammatory transcription factors such as NF-κB, AP-1, and nuclear factor of activated T cells.^33^ NF-κB is one of the best characterized transcription factors, regulating the expression of multiple genes involved in immunity and inflammation. Under basal conditions, NF-κB is localized in the cytoplasm, but once activated it undergoes nuclear translocation allowing DNA binding and increased transcription of inflammatory cytokines, chemokines, adhesion molecules, and inflammatory mediators including inducible nitric oxide synthase (iNOS), ROS, and tumor necrosis factor α (TNFα).^34^ Several studies have reported that nuclear translocation of NF-κB requires PARP-1 function.^35,36^ Following radiation-induced DNA damage, PARP binds to SSBs and recruits BER proteins to induce PARylation and initiate DNA repair. During this process, PARP undergoes automodification which promotes its disassociation from DNA and enables it to form a stable nucleoplasmic protein complex comprised of SUMO1, P1ASγ, NEMO (IKKγ), and ATM. ATM-mediated phosphorylation of NEMO triggers sumoylation of the inactive NF-κB complex in the cytoplasm and subsequent ubiquitination of NEMO from the activated complex. In the nucleus, PARP physically binds to the p65/p50 subunits through p300-CBP histone acetyltransferase, allowing NF-κB-driven transcription of proinflammatory molecules (Figure 2).^37–39^

In vitro and in vivo studies have shown that the response of microglia and astrocytes to inflammation is mediated by PARP-1 and its activation stimulates protein synthesis and proliferation.^9,10,14,34,40–42^ Microglial activation in response to several stimuli including cytokines (eg, TNFα) and alarmins (eg, S100β and HMGB1) is regulated by PARP-1.^34,42,43^ PARP-1 can be activated by DNA damage, usually secondary to reactive oxygen and nitrogen species (ROS/RNS), but also in response to ERK1/2-mediated phosphorylation as a consequence of raised intracellular Ca^2+^.^42,44^

BBB dysfunction is also widely observed in neuroinflammatory-associated diseases. PARP-1 activity is heavily implicated in this phenomenon, with several studies citing associations between PARP activation, edema formation, and increased infiltration of peripheral immune cells into the brain parenchyma.^13,45,46^ Although the exact mechanisms are unclear, it has been proposed that PARP upregulates matrix metalloproteinase-9 (MMP-9) which digests tight junctions and basement membrane proteins, contributing to BBB disruption.^13,47^ PARP-mediated inflammation also increases the expression of adhesion molecules controlling leukocyte migration across the BBB.^45^ Interestingly, PARP activation in monocytes promotes their adhesion to the brain microvascular endothelium and subsequent migration across the BBB by promoting cytoskeletal rearrangements.^25^ PARP-1 also regulates microglia-mediated control of endothelial tight junction integrity influencing BBB permeability.^48^

Finally, PARP-1 is a key mediator of neuronal cell death associated with excitotoxicity, ischemia, and oxidative stress. Hyperactivation of PARP-1 can lead to energy failure due to consumption of NAD^+^ followed by adenosine triphosphate (ATP) depletion that results in cell death and necrosis, promoting further microglia activation.^9^ At the same time, excessive PARP-1 activation also leads to PAR accumulation in the cytoplasm, translocation of apoptosis-inducing factor from the mitochondria to the nucleus and cell death by parthanatos, which is a specific form of cell death that occurs as a result of overactivation of PARP-1.^49^

## PARPi as Anti-inflammatory Agents

As previously mentioned, hyperactivation of PARP can be detrimental, causing neuronal death as well as chronic microglial activation and neuroinflammation. These observations led to the evaluation of PARPi as potential mitigators of neurotoxicity in animal models of CNS pathologies in which neuroinflammation plays a key role.

Many studies have empirically demonstrated protective effects of various PARPi in rodent and primate models of stroke.^50–57^ Ischemia followed by reperfusion appears to be a potent stimulus of PARP overactivation which results in neuronal death, as described above.^56,57^ In addition to reducing necrosis and apoptosis in stroke models, inhibition of PARP has been reported to reduce inflammation, astrogliosis, and microglia activation.^8,58,59^ PARP inhibition decreases proinflammatory cytokines such as interferon gamma (IFNγ), TNFα, IL-6, and IL-17 and increases anti-inflammatory cytokines such as IL-4, IL-10, and transforming growth factor beta 1 (TGFβ1).^60,61^ It also reduces the levels of transcription factors such as NF-κB and inflammatory prostaglandins such as cyclooxygenase 2.^47,61^ Regulatory T cells (Tregs) play a modulatory role in immune responses and can improve outcomes after ischemic strokes. One recent study showed that PARP inhibition upregulates circulating Treg cells, which could help alleviate neuroinflammation by altering cytokine levels.^60^ PARP inhibition has also been shown to protect the vasculature and decrease BBB damage by downregulating VCAM-1, ICAM-1, E-selectin, and MMP-9 expression and reducing extravasation of immunoglobulins.^47,50,61–63^

The neuroprotective properties of PARPi have also been extensively studied in animal models of traumatic brain injury.^9,13,15,64–70^ ROS/RNS produced after the initial trauma cause upregulation of PARP activity leading to secondary additional neuronal injury which can be reversed by PARP inhibition.^69,71^ Apart from the attenuation of early neuronal injury, PARP inhibition in brain trauma models also reduces microglial activation and reactive astrogliosis in rodent and pig (*Sus scrofa*) models.^9,15,72^ As in stroke models, it has been shown that PARP inhibition decreases levels of NF-κB in the cortex, reducing inflammation and downregulating MMP-9 thus preserving BBB function.^13,64^

The role of PARP in neuroinflammatory disease has also been studied in MS and its preclinical model experimental autoimmune encephalitis (EAE), as well as bacterial meningitis.^73–77^ PARP inhibition attenuated oligodendrocyte depletion and decreased demyelination in a rodent model that uses cuprizone.^74^ Of direct clinical relevance, high PARP-1 expression has been identified in blood leukocytes from patients with MS and PARP inhibition was associated with decreased expression of factors such as TGFBR1/TGFBR2/BCL6 in B cells.^73^ As in stroke models, the immunomodulatory effects of PARPi appear to involve both increased numbers of Tregs and enhanced function.^76^ Consistent with these findings, expression of ICAM-1, NF-κB, and the inflammatory mediators interferon-γ, TNFα, and iNOS were decreased in CNS tissues from rodent models treated with PARPi.^75,76^

Evidence supporting important pathogenic roles for PARP-1 now extends to neurodegenerative diseases, in which it has been observed to promote neuronal cell death, mitochondrial function, and neuroinflammation. Preclinical studies have shown therapeutic benefits of a variety of PARPi in rodent models of PD, AD, and Huntington’s disease.^78–82^ Reduced microglial activation in response to PARP inhibition has been reported in all these diseases.^78,80,82^

Much less evidence is available in the context of radiation-induced neuroinflammation. However, the intracellular mechanisms implicated in this phenomenon share many of the same signaling pathways as the CNS diseases described above, and there is a prominent inflammatory component. As described earlier, radiation has been shown to regulate NF-κB transcription factors in the brain, causing microglial activation and increasing transcription of inflammatory cytokines, chemokines, adhesion molecules, and inflammatory mediators including iNOS, ROS, and TNFα. All these factors are implicated in the many neurological conditions in which PARP inhibition has shown promising results. In this context, it seems entirely reasonable to propose that PARP inhibition could play a neuroprotective role in suppressing radiation-induced neuroinflammation and thus reducing the associated cognitive decline.

## Widening the Therapeutic Ratio by Combining PARPi With Radiation

As previously mentioned, PARP-1 plays an important role in DNA damage repair, predominantly by stimulating and facilitating function of the BER pathway, and with additional roles in alternative NHEJ, HR, and replication fork restart.^83,84^ In keeping with these roles, PARP inhibition causes accumulation of DNA breaks in replicating cells and PARPi have potent single agent activity against cells harboring defects in HR repair.^85^ Of greater relevance to their potential roles in neuro-oncology, PARPi also exhibit potentiating effects when combined with DNA damaging agents such as TMZ and RT, which together form “standard of care” for GBM patients. Multiple studies in preclinical models have demonstrated that PARPi potently augment the cytotoxic effects of TMZ and consistently enhance radiosensitivity across a broad range of cancer types including GBM. Importantly, these radiosensitizing effects are only observed in proliferating cells, in which unresolved SSBs are converted into DSBs during replication, thus enhancing the cytotoxic effects of radiation.^86^ In the context of GBM, therefore, PARP inhibition is predicted to enhance the capacity of RT to ablate rapidly proliferating tumor cells while having minimal effect on cells in the surrounding normal brain, which are almost exclusively nonreplicating.

Until recently, one of the main limitations to evaluating PARPi in the treatment of brain tumors concerns their ability to penetrate the BBB. Some newly developed PARPi such as pamiparib (BeiGene) and niraparib (Zejula, Tesaro) have shown improved BBB penetration in mice, making them attractive options.^87,88^ However, a recent phase I clinical trial reported that olaparib (Lynparza, AstraZeneca) penetrates both tumor core and margin regions in patients with recurrent GBM, despite failing to penetrate the BBB of mice and rats. These findings support the concept that the BBB is sufficiently disrupted in and adjacent to GBM that even “nonbrain penetrant” PARPi may have clinical activity.^89^

Several studies using different PARPi provide strong evidence of radiosensitizing activity both in vitro and in vivo in a variety of preclinical models of adult and pediatric glioma.^86,90–94^ The radiosensitizing effects of PARPi have also been reported in other CNS tumors such as medulloblastoma and ependymoma.^90^

These preclinical data underpin multiple phase I/II clinical trials that have been completed or are actively recruiting, evaluating 3 different PARPi (olaparib [Lynparza, AstraZeneca], veliparib [AbbVie], and pamiparib [BeiGene]) in combination with RT and/or TMZ in patients with gliomas, predominantly but not exclusively GBM. A comprehensive list of the current clinical trials can be found in a recent review.^16^

**Figure 3.**
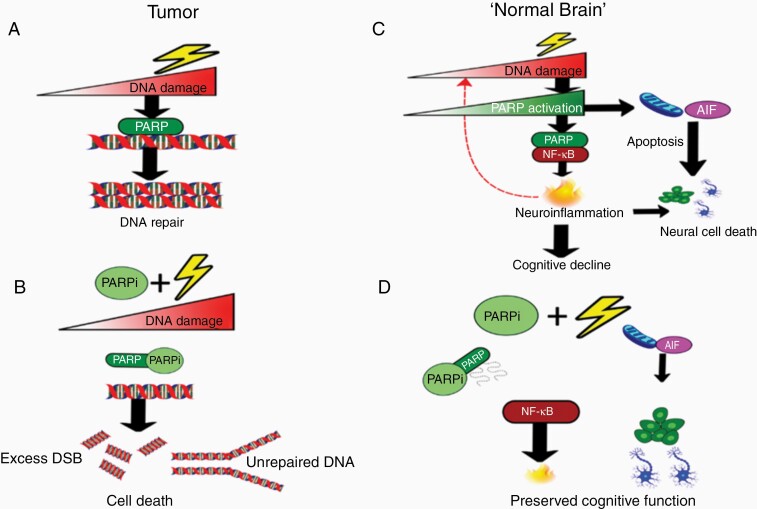
Differential impact of radiation exposure ± PARP inhibition in tumor and normal brain. (A) In tumor cells, PARP binds to DNA SSB in glioma cells and through the recruitment of BER proteins, facilitates DNA repair. (B) PARP inhibitors compete with NAD^+^ at the PARP catalytic domain causing inhibition of PARP catalytic activity and the accumulation of unrepaired SSBs. This leads to the formation of DSBs, increased genomic instability, and cell death. (C) In the normal brain, radiation-induced DNA damage and the associated increase in PARP hyperactivity through interaction with NF-κB results in neuroinflammation, leading to reciprocal DNA damage and neural cell death. Hyperactivation of PARP and neuroinflammation can also exacerbate PARP-dependent cell death through the nuclear transfer of AIF from mitochondria, leading to apoptotic cell death of neurogenic cells contributing to cognitive decline. (D) PARP inhibitors prevent PARP activation and PARP’s interaction with NF-κB, causing a reduction in the expression of inflammatory mediators and a reduction in neuroinflammation. Additionally, reduced PARP activation mitigates the onset of PARP-dependent cell death of neuronal cells. AIF, apoptosis-inducing factor; BER, base excision repair; DSBs, double strand breaks; NAD^+^, nicotinamide adenine dinucleotide; NF-κB, nuclear factor κB; PARP, poly(ADP-ribose) polymerase; SSBs, , single strand breaks.

Taking all this clinical and preclinical together, we propose that the strategy of combining RT and PARP inhibition for the treatment of GBM holds exceptional promise for this cancer of extreme unmet need. Tumor cell death will be enhanced by inhibition of DNA repair pathways, whilst normal brain tissue may be protected both from radiation-induced neuroinflammation, via suppression of glial activation and inflammatory mediators, and from radiation-driven apoptosis of NPCs and neurons, via avoidance of ATP depletion (Figure 3).

Having made this proposal, we would also like to acknowledge the crucial role played by the tumor microenvironment in influencing treatment responses of GBM and other brain tumors, and the additional layers of complexity that this creates. Tumor-associated macrophages and microglia have been shown to play an important role in creating an immunosuppressive tumor environment that protects tumor cells from recognition and destruction by the immune system.^95^ While one might speculate that PARP inhibition would further suppress microglial activity, thus protecting tumor cells, a recent abstract described the opposite effect.^96^ This illustrates the importance of assessing the neuroinflammatory effects of PARP inhibition in microenvironments that recapitulate the clinical scenario. More specifically, the presence of a tumor might alter the immunomodulatory effects of both radiation and PARP inhibition. Adding further complexity, combining DNA damaging agents such as radiation with PARPi has potential to enhance the immunogenicity of the tumor cells by promoting neoantigen release, increasing tumor mutational burden, and enhancing immunogenic signaling via the cGAS-STING pathway.^97^

Considering these complex interactions, we would like to emphasize the urgent need to develop new, orthotopic, immunocompetent models of GBM that would allow detailed study of the multifaceted effects of PARP inhibition and their consequences on cell survival, neuroinflammation, and cognition in the context of a tumor bearing brain with an intact immune system.

While this review focuses on PARP, it is important to mention that other proteins of the DDR pathway, including ATM, ataxia–telangiectasia, and Rad3-related protein (ATR) and checkpoint kinase 1 (CHK1) have also shown radiosensitizing effects in preclinical models of GBM.^98,99^ ATM inhibitors are potent tumor radiosensitizers that are currently being evaluated in clinical trials in GBM patients; they may have neuroprotective effects by modulating oxidative stress and preventing neuronal apoptosis.^98,100^ ATR and CHK1 exert modest radiosensitizing effects similar to PARPi.^98^ Neither ATM, ATR, nor CHK1 inhibitors have been shown to regulate neuroinflammation.

## Conclusions

Although, our understanding of the pathophysiology of radiation-induced cognitive dysfunction is currently limited, there is strong evidence supporting a major role of neuroinflammation. Recent data have identified neuroinflammation as a common feature in many CNS diseases including brain trauma, stroke, and multiple neurodegenerative processes, and preclinical studies have provided robust evidence to support the use of PARPi to alleviate neuroinflammation in these conditions. At the same time the use of PARPi as radiosensitizers for the treatment of brain tumors has generated promising preclinical data and multiple early phase clinical trials are ongoing. This, in combination with the availability of brain penetrant PARPi, offers the intriguing opportunity to radiosensitize brain tumor cells, while simultaneously reducing radiation-induced neuroinflammation. The translation of these exciting preclinical findings to the clinic offers the promise of improving survival and quality of life for brain tumor patients receiving RT. It will be important to include neuroinflammatory markers, cognitive function scores, and quality of life measures when designing future clinical trials, to help elucidate the potential neuroprotective role of PARPi when combined with radiation.
